# SUMOylation of PES1 upregulates its stability and function via inhibiting its ubiquitination

**DOI:** 10.18632/oncotarget.10494

**Published:** 2016-07-08

**Authors:** Shujing Li, Miao Wang, Xinjian Qu, Zhaowei Xu, Yangyang Yang, Qiming Su, Huijian Wu

**Affiliations:** ^1^ School of Life Science and Biotechnology, Dalian University of Technology, Dalian, China; ^2^ School of Life Science and Medicine, Dalian University of Technology, Panjin, China

**Keywords:** PES1, SUMOylation, breast cancer, ubiquitination

## Abstract

PES1 is a component of the PeBoW complex, which is required for the maturation of 28S and 5.8S ribosomal RNAs, as well as for the formation of the 60S ribosome. Deregulation of ribosomal biogenesis can contribute to carcinogenesis. In this study, we showed that PES1 could be modified by the small ubiquitin-like modifier (SUMO) SUMO-1, SUMO-2 and SUMO-3, and SUMOylation of PES1 was stimulated by estrogen (E2). One major SUMOylation site (K517) was identified in the C-terminal Glu-rich domain of PES1. Substitution of K517 with arginine abolished the SUMOylation of PES1. SUMOylation also stabilized PES1 through inhibiting its ubiquitination. In addition, PES1 SUMOylation positively regulated the estrogen signaling pathway. SUMOylation enhanced the ability of PES1 to promote estrogen receptor α (ERα)-mediated transcription by increasing the stability of ERα, both in the presence and absence of E2. Moreover, SUMOylation of PES1 also increased the proportion of S-phase cells in the cell cycle and promoted the proliferation of breast cancer cells both *in vitro* and *in vivo*. These findings showed that posttranslational modification of PES1 by SUMOylation may serve as a key factor that regulates the function of PES1 in vivo.

## INTRODUCTION

PES1 is a nuclear protein discovered in 1996, and early studies have shown that several abnormal developments that occurred in the brain, eye, liver and other organs of zebrafish embryo as a result of PES1 mutation can even cause the death of the embryo [1, 2]. The human *PES1* gene, which is located to 22q12.1 was first cloned in 2001 [3]. The PES1 protein contains 588 amino acids and it has three nuclear localization signals (NLSs) that mediate the transport of the protein into the nucleus. PES1 is mainly localized in the nucleolus, and rarely distributed in the cytoplasm. The C terminus (amino acids 315-412) of PES1 contains a typical BRCT domain that has been found in the carboxyl terminus of one breast cancer suppressor protein, BRCA1. Many proteins containing the BRCT domain play a role in DNA repair, remodeling and cell cycle. The BRCT domain of PES1 plays a crucial role in regulating the progress of cell cycle [4]. In addition, PES1 also contains a glutamate rich region, but its function is not clear.

The main function of PES1 is its involvement in the synthesis and maturation of ribosome and its effect on chromatin stretch. More and more research have shown that PES1 is closely associated with tumorgenesis via improving cell proliferation and participating in the progress of cell cycle in cancer cells. PES1 is mainly expressed in developing tissues, but aberrant expression of PES1 has been found in human brain tumors. Neuroblastoma (NB) cases with MYCN amplification and international neuroblastoma staging system stage 4 (INSS stage 4) tend to show a higher expression level of PES1 [5]. In addition, high PES1 expression is associated with the worst overall and relapse-free survival [5]. PES1 is also upregulated in several different carcinoma cell lines, such as the colon cancer cell line SW480 and human breast cancer cell line MCF-7 [6]. PES1 is upregulated more in colon cancer tissues than in non-cancerous tissues, while down-regulation of PES1 in colon cancer cells can lead to cell cycle arrest at the G1 phase, as well as reduced proliferation and decreased growth of xenografts [7]. Clinical study has suggested that the expression of PES1 in breast cancer tissues (stage I-IV is much higher than that in normal breast tissues, while PES1 knockdown represses the growth and tumorgenesis of breast cancer cells [8]. One possible mechanism by which PES1 promotes the development of breast cancer (ERα-positive) is through increasing the level of ERα while decreasing the level of ERβ [9]. ERα promotes the stimulating effect of estrogen on breast cancer cell proliferation, whereas ERβ inhibits the proliferation of breast cancer cells. All these data show that abnormality associated with PES1 may be a common feature of malignancy.

Small ubiquitin like-modifier (SUMO) modification is a crucial post-translational modification for regulating protein activity, stability, alteration of protein-protein interaction and transcriptional activity as well as protein sublocalization in the cells. A consensus sequence ΨKXE exists in most proteins that are modified by SUMOylation. In this sequence, Ψ stands for a hydrophobic amino acid and K is the conjugation site for SUMO. SUMOylation modification is a dynamic process, in which the modified proteins can be deSUMOylated by SUMO-specific proteases (SENPs) [10]. Many proteins with important roles in cellular processes have been identified as targets that are modified by SUMO. For examples, SUMOylation of DEC1 increases its ability to repress the transcriptional activity of CLOCK/BMAL1 [11], and SUMOylation of GPS2 promotes its ability to inhibit ERα-mediated transcription [12]. Sequence analysis of PES1 revealed two potential SUMO conjugation sites, K249 and K517, both of which were contained within the consensus sequence ΨKXE, so we wanted to examine whether PES1 is a target protein of SUMO.

In this study, we demonstrated that PES1 could be SUMOylated by SUMO-1, −2 and −3, and showed that K517 was the main SUMOylation site of PES1. SUMOylation stabilized PES1 byinhibiting its ubiquitination. PES1 SUMOylation also promoted the transcriptional activity of ERα by enhancing its stability. SUMOylation of PES1 inhibited the association of ERα with its ubiquitination E3 ligase ChIP and as well as inhibiting the ubiquitination of ERα. Moreover, SUMOylation of PES1 increased the percentage of MCF-7 cells in the S phase and promoted the proliferation of breast cancer cells as well as the formation of xenograft tumor. Taken together, our data suggested that post-translation modification of PES1 via SUMOylation might play important roles in ERα-related estrogen signaling pathway and in the growth of breast cancer cells.

## RESULTS

### PES1 is modified by SUMO

**Figure 1:**
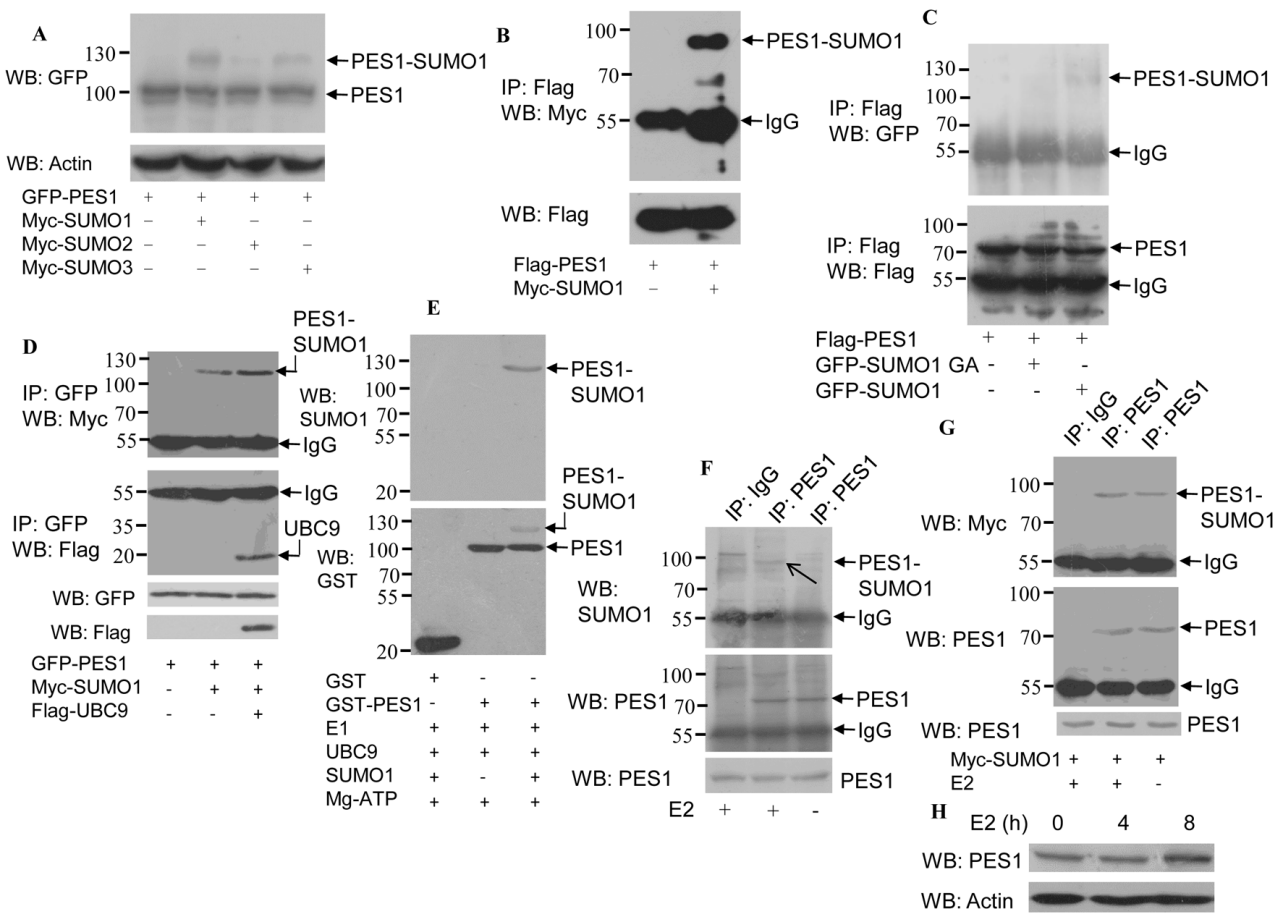
Modification of PES1 by SUMO-1, SUMO-2 and SUMO-3 **A.** COS-7 cells were co-transfected with Myc-SUMO-1, −2 or −3 and GPF-tagged PES1, and then subjected to western blot with anti-GFP antibody. **B.** COS-7 cells were transfected with Flag-tagged PES1 and Myc-tagged SUMO1 and then subjected to immunoprecipitation with anti-Flag antibody followed by western blot with anti-Myc antibody. **C.** COS-7 cells were cotransfected with Flag-tagged PES1 and GFP-tagged SUMO1 or SUMO1/GA and then subjected to immunoprecipitation with anti-Flag antibody, followed by western blot with anti-GFP antibody. **D.** COS-7 cells were transfected with GFP-PES1 alone, GFP-PES1 plus Myc-SUMO1 without or with UBC9, and then subjected to immunoprecipitation with anti-GFP antibody followed by western blot with anti-Myc or anti-Flag antibody. **E.** In vitro SUMOylation assay was performed using a SUMOylation kit (Enzo Life Sciences). Affinity-purified GST or GST-PES1 was incubated with SAE1-SAE2 (E1), UBC9 (E2), Mg-ATP and SUMO-1, as indicated at 30°C for 60 min, and then analyzed by immunoblotting. **F.** MCF-7 cells were subjected to serum starvation for 3 days, followed by treatment with 10 nM E2 for 4 h, and the cell extract was then subjected to immunoprecipitation with anti-PES1 antibody or anti-immunoglobulin G, followed by western blot analysis with anti-SUMO1 or anti-PES1 antibody. **G.** MCF-7 cells were transfected with Myc-SUMO1 for 24 h, and the cells were then subjected to serum starvation for 3 days, followed by treatment with 10 nM E2 for 4 h. Cell extract was prepared and subjected to immunoprecipitation with anti-PES1 antibody or anti-immunoglobulin G, followed by western blot analysis with anti-Myc or anti-PES1 antibody. **H.** MCF-7 cells were starved for 3 days, followed by treatment with 10 nM E2 for different periods, and then subjected to western blot analysis with anti-PES1 antibody.

To identify whether PES1 is a SUMOylated substrate, COS-7 cells were cotransfected with GFP-PES1 and Myc-SUMO-1, −2 or −3. Western blot analysis of PES1 showed a band with a molecular mass much higher than that of PES1 when PES1 was coexpressed with SUMO-1, −2 or −3 (Figure 1A), suggesting that PES1 was modified by all three kinds of SUMO. Since PES1 was mainly modified by SUMO-1, we therefore focused mainly on the functions of PES1 modified by SUMO-1 in subsequent experiments. Moreover, analysis of PES1 SUMOylation in COS-7 cells cotransfected with Flag-PES1 and Myc-SUMO-1 showed that the overexpressed PES1 was modified by SUMO-1 (Figure 1B). To further confirm the SUMOylation of PES1, we cotransfected COS-7 cells with Flag-PES1 and GFP-SUMO-1 or GFP-SUMO-1 GA and analyzed the SUMOylated products. SUMO-1 GA is a SUMO mutant that lacks the ability to join with the substrate due to a C-terminal diglycine substitution (GG to GA). A band of higher molecular mass indicative of PES1-SUMO1 was detected only when PES1 was coexpressed with wild-type SUMO-1 in the cells (Figure 1C). The effect of UBC9, a SUMO E2 conjugate enzyme, on the SUMOylation of PES1 was also examined. Cells transfected with UBC9 and SUMO-1 showed the highest intensity for the PES1-SUMO-1 band (Figure 1D). In addition, a SUMOylation assay conducted with purified recombinant SUMO-conjugating enzymes and GST-PES1 clearly showed that PES1 could be modified by SUMO-1 *in vitro* (Figure 1E). The effect of estrogen (E2) on endogenous SUMOylated PES1 was also investigated. PES1 immunoprecipitated from MCF-7 cells was detected by anti-SUMO1 antibody, and its level increased when the cells were treated with E2 (Figure 1F). To further confirm this result, MCF-7 cells were transfected with Myc-SUMO-1, and PES1 was then immunoprecipitated from the cells and probed with anti-Myc antibody. The result showed that the intensity of the band corresponding to SUMOylated PES1 increased with E2 treatment (Figure 1G). The protein level of PES1 was upregulated when the E2 treatment lasted for 8 h, but remained unchanged when the E2 treatment lasted for 4 h (Figure 1H). These data suggested that PES1 could be modified by SUMO, and E2 may upregulate the SUMOylation of PES1.

### K517 is the primary site for PES1 SUMOylation

**Figure 2:**
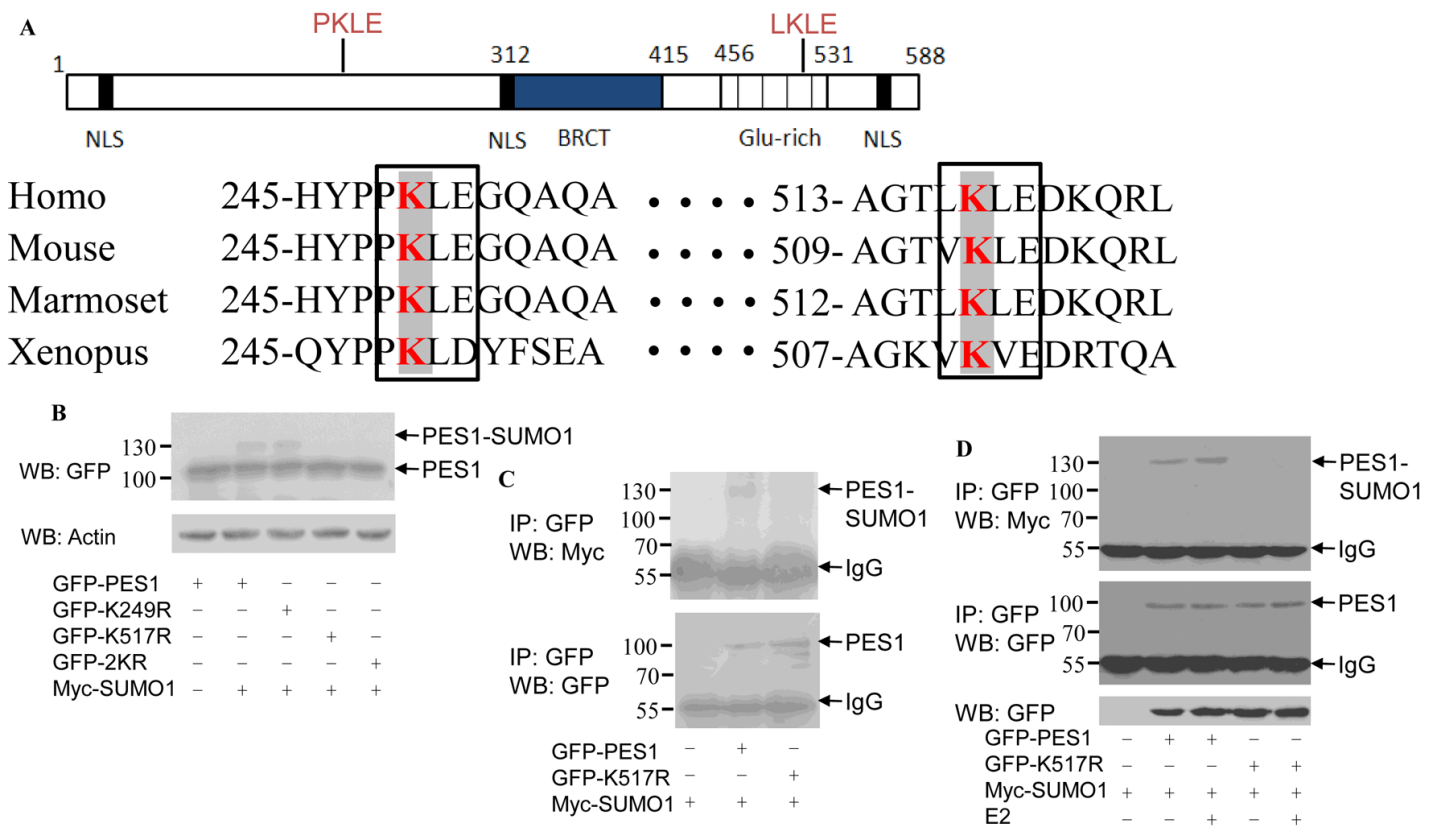
Identification of SUMOylation sites in PES1 **A.** Schematic representation of human PES1, showing the conserved lysine residues within the Glu rich region. The amino acids surrounding the two conserved lysine residues were compared with those of PES1 from three other species: mouse, Marmoset and Xenopus. Conserved lysine residues are boxed. **B.** COS-7 cells were transfected with wild-type GFP-PES1 or its mutants K249R, K517R or K249R/K517R (2KR) and Myc-tagged SUMO-1, and then subjected to western blot with anti-GFP antibody. **C.** COS-7 cells were transfected with wild-type GFP-PES1 or its mutant K517R as well as Myc-SUMO-1, and then subjected to immunoprecipitation with anti-GFP antibody followed by western blot with anti-Myc or anti-GFP antibody. **D.** MCF-7 cells were transfected with wild-type GFP-PES1 or its mutant K517R as well as Myc-SUMO-1 for 24 h, and the cells were then subjected to serum starvation for 3 days, followed by treatment with 10 nM E2 for 4 h. Cell extract was then prepared and subjected to immunoprecipitation with anti-GFP antibody, followed by western blot analysis with anti-Myc or anti-GFP antibody.

Analysis of the sequence of PES1 revealed two conserved lysine residues in the Glu-rich domain: one corresponding to K249 and the other to K517 (Figure 2A). Furthermore, these two sites are conserved in different species (homo, mouse, marmoset and xenopus) (Figure 2A). Changing K249 to arginine (K249R) resulted in nearly no change in the SUMOylation status of PES1, whereas changing K517 to arginine (K517R) or both K249 and K517 to arginine (2KR) appeared to abolish the SUMOylation of PES1 (Figure 2B). To further define the SUMOylation sites in PES1, COS-7 cells were transfected with Myc-SUMO1 together with GFP-PES1 or GFP-K517R. GFP-PES1 immunoprecipitated from the cell extract was detected by anti-Myc antibody, while the immunoprecipitated GFP-K517R did not show any reaction with the anti-Myc antibody, indicating that the SUMO-1 did not conjugate with the mutant PES1 (Figure 2C). The effect of E2 on the SUMOylation of the mutant PES1 was also examined. E2 slightly increased the SUMOylation of wild-type PES1, but had no effect on the SUMOylation of the mutant PES1 (Figure 2D). These data indicated that K517 of PES1 could be the key SUMOylation site.

### Effect of SUMOylation on the localization and stability of PES1

**Figure 3:**
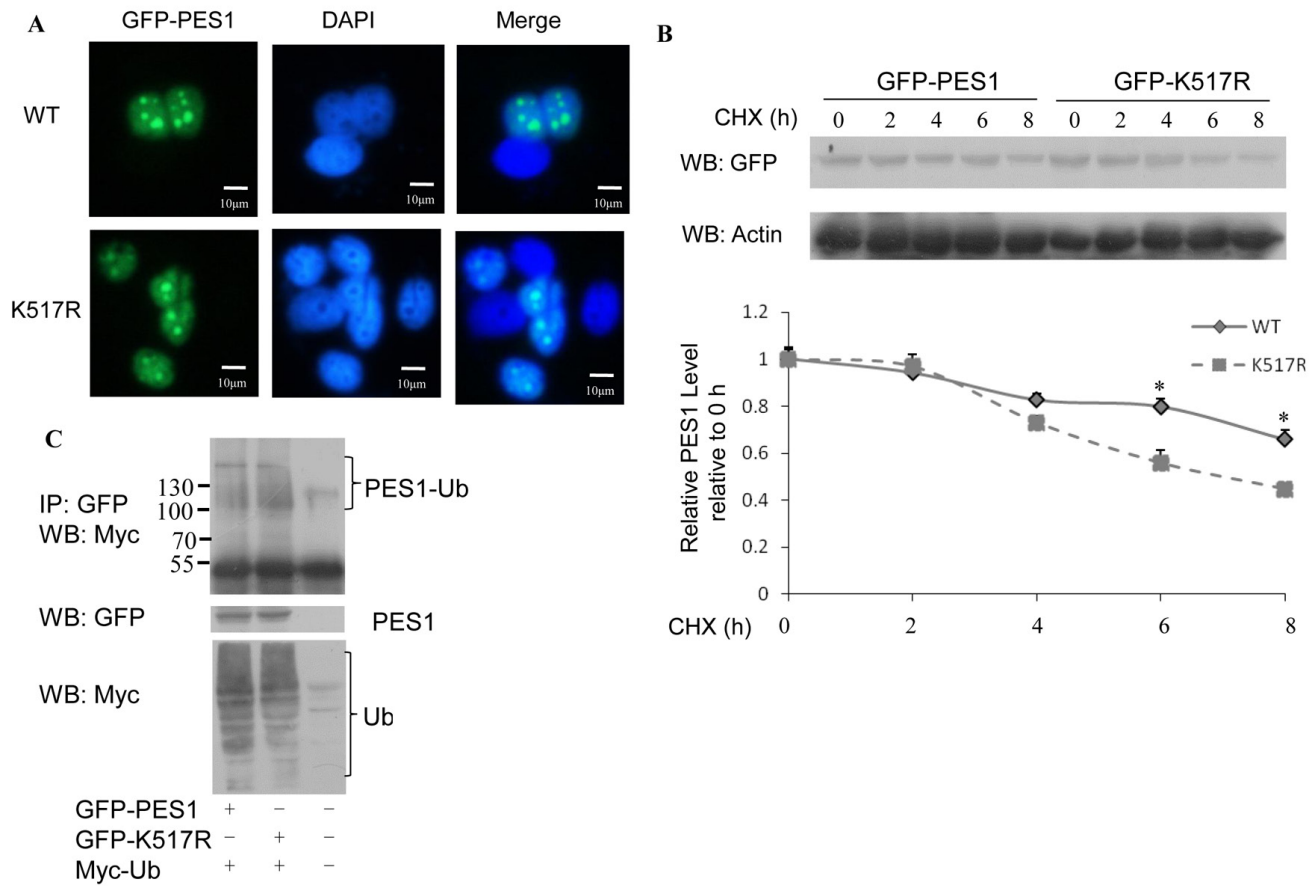
Effect of SUMOylation on the nucleolar distribution and stability of PES1 **A.** MCF-7 cells transfected with GFP-tagged wild-type PES1 or mutant PES1 K517R were stained with GFP. Nuclei were stained with 4,6-diamidino-2-phenylindole (DAPI). **B.** MCF-7 cells were transfected with GFP-tagged wild-type PES1 or K517R and treated with CHX at the indicated time periods. Cell lysate was subjected to western blot with anti-GFP antibody. **C.** MCF-7 cells were transfected with GFP-tagged wild-type PES1 or K517R together with Myc-tagged ubiquitin, and then treated with MG132, a proteasome inhibitor, for 6 h before harvest. Cell lysate was subjected to immunoprecipitation with anti-GFP antibody followed by western blot with anti-Myc antibody.

As reported in previous study [13], PES1 is localized in the nuclei of AR5 cells (human fibroblast cell line). SUMOylation is considered to influence the functions of proteins by changing their properties such as subcellular localization and protein stability. We speculated that SUMOylation might affect the subcellular localization of PES1. To test this speculation, we transfected MCF-7 cells with GFP-tagged PES1 or its mutant K517R and the cells were then subjected to immunofluorescence assay. The result showed that PES1 was mainly localized in the nucleus of the cells, regardless of whether it was wild-type or mutant PES1 (Figure 3A). This indicated that SUMOylation might not influence the subcellular localization of PES1.

The effect of SUMOylation on PES1 protein stability was examined by the half-life assay. The half-life of wild-type PES1 was more than 8 h, while the half-life of the mutant was between 6 h and 8 h (Figure 3B). This indicated that SUMOylation stabilized the protein level of PES1. Since the stability of a protein may be subject to regulation by ubiquitination, it became necessary to examine whether the increased stability of SUMOylated PES1 was due to suppression of its ubiquitination. Ubiquitination wild-type PES1 was reduced compared to the mutant (Figure 3C), showing that SUMOylation of PES1 repressed its ubiquitination.

### PES1 K517R promotes Trim23-mediated PES1 ubiquitin-proteasome pathway

**Figure 4:**
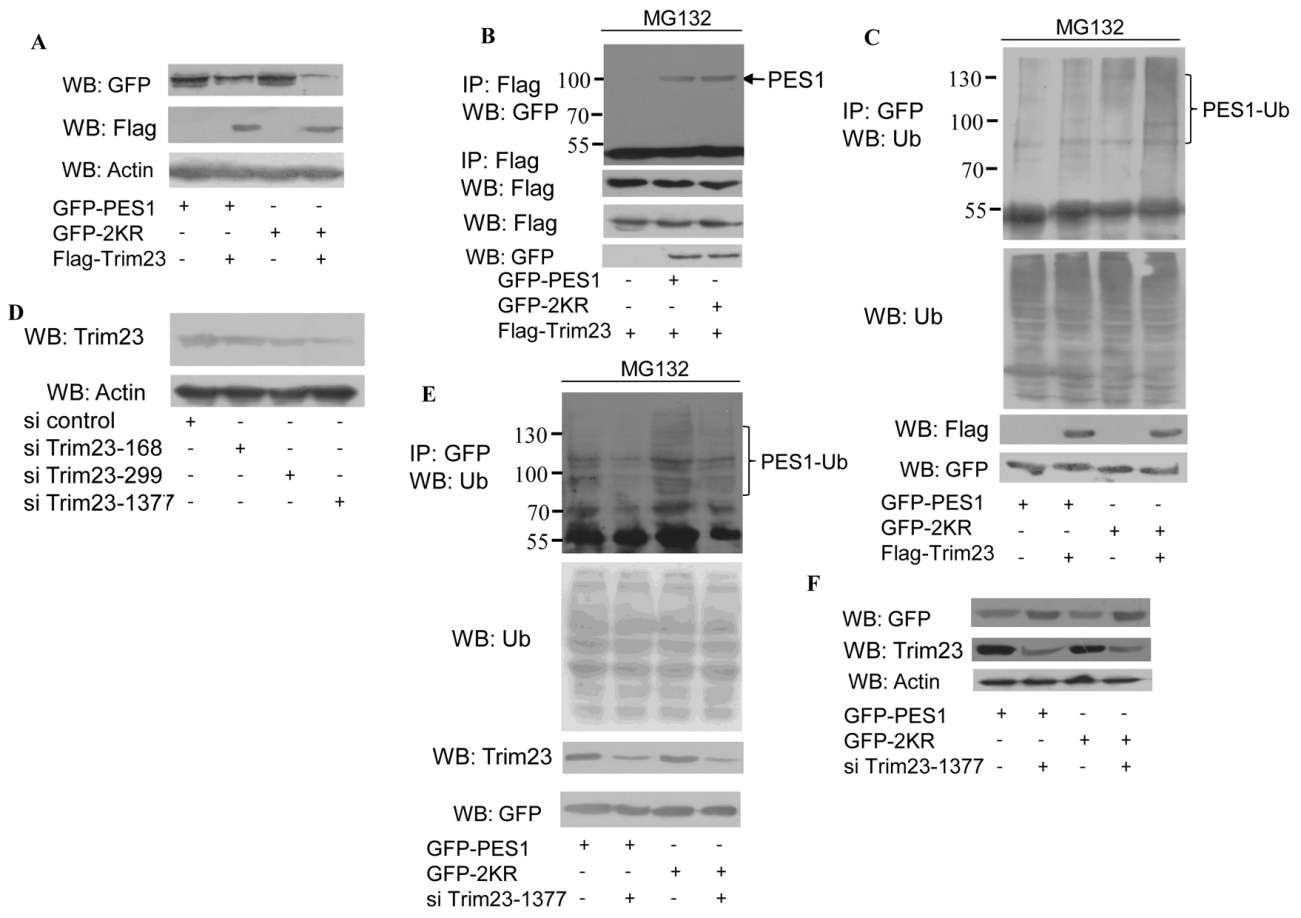
Effect of ubiquitin E3 ligase Trim23 on the stability of PES1 **A.** COS-7 cells were transfected with GFP-tagged wild-type PES1 or mutant PES1 K517R together with Flag-Trim23 or not, and then subjected to western blot with anti-GFP antibody. **B.** COS-7 cells were transfected with Flag-tagged Trim23 plus GFP-tagged wild-type PES1 or K517R, and then treated with MG132, a proteasome inhibitor, for 6 h before harvest. Cell lysate was subjected to immunoprecipitation with anti-Flag antibody followed by western blot with anti-GFP antibody or anti-Flag antibodies. **C.** COS-7 cells were transfected with GFP-tagged wild-type PES1 or K517R without or with Trim23, and then treated with MG132, a proteasome inhibitor, for 6 h before harvest. Cell lysate was subjected to immunoprecipitation with anti-GFP antibody followed by western blot with anti-ubiquitin antibody. **D.** MCF-7 cells transfected with si control, si Trim23-168, si Trim23-299 or si Trim23-1377 were subjected to western blot with anti-Trim23 antibody. **E.** MCF-7 cells were transfected with GFP-PES1 or GFP-PES1 K517R together with or without si-Trim23-1377. The cell extract was subjected to immunoprecipitation with anti-GFP antibody, followed by western blot using anti-Ub antibody. **F.** MCF-7 cells were transfected with GFP-PES1 or GFP-PES1 K517R together with or without si-Trim23-1377, and then subjected to western blot with anti-GFP antibody.

To further study the role of SUMOylation in the regulation of PES1 stability, the effects of several ubiquitin E3 ligases were investigated. PES1 level was most significantly affected by Trim23 (data not shown). COS-7 cells transfected with Trim23 plus GFP-tagged wild-type PES1 or mutant PES1 (K517R) showed reduced protein stability with respect to PES1, but the level of K517R was much lower than that of wild type (Figure 4A). Co-immunoprecipitation showed that the interaction between wild-type PES1 and Trim23 was noticeably weaker than the interaction between K517R and Trim23 (Figure 4B). In addition, the ubiquitination of PES1 K517R was increased compared to that of wild-type PES1, especially in the presence of Trim23 (Figure 4C). On the other hand, the ubiquitination of PES1 was decreased when Trim23 was knockdown (Figure 4E) (Si Trim23-1377 appeared to be more effective than the other two shown in Figure 4D). The protein level of PES1 (both wild type and K517R) was also increased with the knockdown of Trim23 (Figure 4F). Taken together, the results indicated that SUMOylation of PES1 may inhibit its interaction with Trim23, resulting in increased stability for PES1.

### Effect of SUMOylation on protein stability of ERα

**Figure 5:**
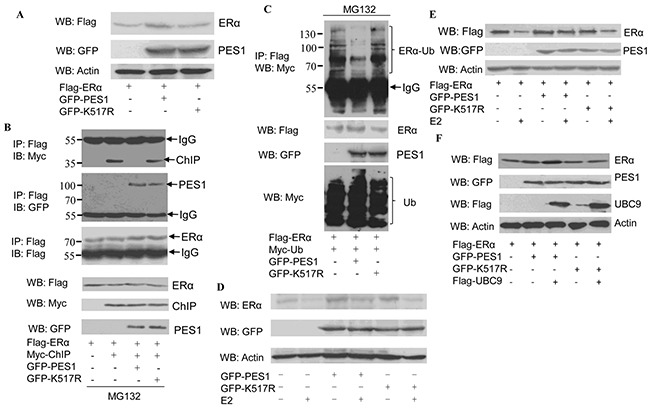
Effect of PES1 SUMOylation on the stability of ERα **A.** COS-7 cells were transfected with Flag-ERα together with GFP-tagged wild-type PES1 or K517R, and then subjected to western blot with anti-Flag antibody. **B.** COS-7 cells were transfected with Flag-ERα and control vector or Myc-ChIP together with GFP-tagged wild-type PES1 or K517R, and then treated with MG132, a proteasome inhibitor, for 6 h before harvest. Cell lysate was subjected to immunoprecipitation with anti-Flag antibody followed by western blot with anti-Myc, anti-GFP or anti-Flag antibodies. **C.** COS-7 cells were transfected with Flag-ERα and Myc-Ub together with GFP-tagged wild-type PES1 or K517R, and then treated with MG132 for 6 h before harvest. Cell lysate was subjected to immunoprecipitation with anti-GFP antibody followed by western blot with anti-Myc antibody. **D.** MCF-7 cells were transfected with GFP-tagged wild-type PES1 or K517R for 24 h. The cells were starved for 3 days, and then treated with or without 10 nM E2 for 24 h. The cell extract was subjected to western blot with anti-ERα. **E.** MCF-7 cells were transfected with Flag-ERα together with GFP-tagged wild-type PES1 or K517R for 24 h. The cells were starved for 3 days, and then treated with or without 10 nM E2 for 24 h. The cell extract was subjected to western blot with anti-Flag antibody. **F.** COS-7 cells were transfected with Flag-ERα, GFP-tagged wild-type PES1 or K517R together with or without Flag-UBC9, and then subjected to western blot with anti-Flag antibody.

PES1 promotes the growth of breast cancer cells by improving the stability of ERα [9]. Overexpression of wild-type PES1 in COS-7 cells increased the protein level of ERα while overexpression of K517R caused no change to the level of ERα (Figure 5A). PES1 promotes the stability of the ERα through inhibiting the interaction between ERα and the carboxyl terminus of Hsc70-interacting protein (CHIP), and subsequently repressing the ubiquitin-proteasome pathway-mediated degradation of ERα [14]. Next we examined whether PES1 SUMOylation plays a role in regulating the interaction between ERα and CHIP. Interaction between ERα and CHIP was not detected in the extract of the cells that overexpressed wild-type PES1, but was detected in the extract of the cells that overexpressed the mutant PES1 or did not overexpress PES1 (Figure 5B). This data demonstrated that SUMOylation of PES1 inhibited the interaction between CHIP and ERα. Moreover, the role of PES1 SUMOylation in the ubiquitination of ERα was also examined. Overexpression of wild-type PES1 decreased the ubiquitination of ERα compared to the control (no overexpression of PES1), while the mutant PES1 K517R had a weaker effect on the ubiquitination of ERα compared to wild-type PES1 (Figure 5C). In addition, PES1-mediated ERα stability was not affected by E2, although wild-type PES1 conferred a higher degree of stability to ERα than K517R. (Figure 5D and 5E). Overexpression of UBC9 together with wild-type PES1 further promoted the stability of ERα (Figure 5F). These data showed that the stability of ERα promoted by PES1 was dependent on its SUMOylation.

### SUMOylation of PES1 promoted the transcriptional activity of ERα

**Figure 6:**
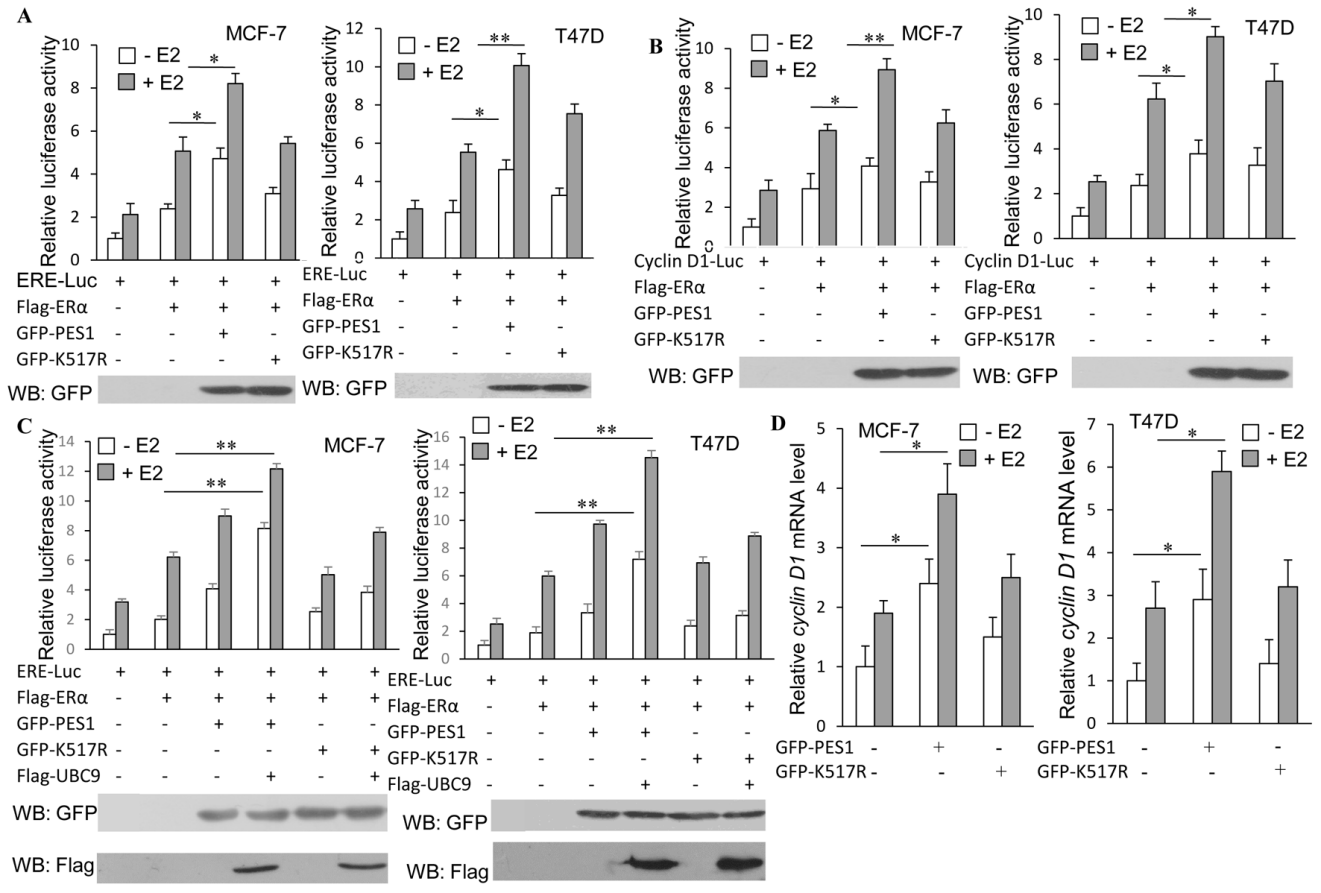
SUMOylation of PES1 upregulates the transcriptional activities of ERα and its downstream target gene **A.** MCF-7 or T47D cells were transfected with ERE-Luc or with ERα, or with ERα and GFP-tagged wild-type PES1 or K517R. Luciferase activity was measured either with or without pre-treatment of the cells with 10 nM E2 for 16 h. **B.** Similar experiment as described in A was performed, but the cells were transfected with Cyclin D1-luciferase. **C.** MCF-7 or T47D cells were transfected with ERE-Luc or with ERα, or with ERα and GFP-tagged wild-type PES1 or K517R together with or without Flag-UBC9. Luciferase activity was measured either with or without pre-treatment of the cells with 10 nM E2 for 16 h. **D.** MCF-7 or T47D cells were transfected with wild-type PES1 or K517R. The cells were pre-treated with or without 10 nM E2 for 16 h and then subjected to RT-PCR to measure the mRNA level of *Cyclin D1*. The mRNA level of *cyclin D1* was expressed relative to *GAPDH* transcriptional level. ‘*’, *p*<0.05; ‘**’, *p*<0.01.

To examine whether SUMOylation of PES1 also affected the transcriptional activity of ERα, luciferase reporter assays were performed. As anticipated, the highest level of luciferase activity was found in cells that overexpressed both ERα and wild-type PES1, whether with or without E2 treatment, while overexpression of K517R reduced its ability to activate the transcriptional activity of ERα (Figure 6A). The effect of PES1 SUMOylation on the regulation of *cyclin D1* expression, a well established ERα-target gene, was also examined. Wild-type PES1 promoted the expression of *cyclin D1* in the presence or absence of E2, but this effect of PES1 was compromised when the SUMOylation site was mutated (Figure 6B). Overexpression of UBC9 together with wild-type PES1 further promoted the activity of ERE-Luc reporter in MCF-7 and T47D cells (Figure 6C). Similar result was also obtained from RT-PCR analysis, where a higher level of *cyclin D1* mRNA was found in the cells transfected with wild-type PES1 than in the cells transfected with the mutant PES1 (Figure 6D). All these data showed that PES1 SUMOylation had a crucial effect on the regulation of ERα activity.

### Effect of PES1 SUMOylation on breast cancer cell lines proliferation

**Figure 7:**
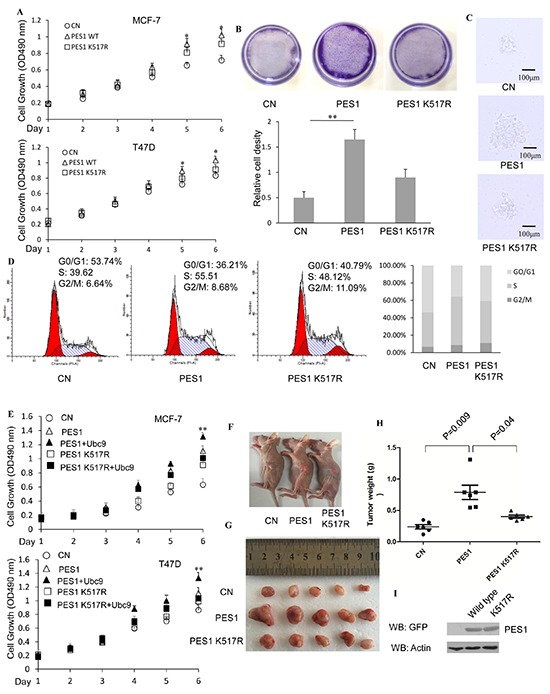
SUMOylation of PES1 promotes the growth of breast cancer cell lines **A.** MCF-7 or T47D cells were stably transfected with GFP-tagged PES1 or K517R. The cells were grown in the presence of E2 and then subjected to MTT assay performed according to the manufacturer's instructions. **B.** Crystal violet staining (MCF-7 cells). The cells were treated with E2 for 7 days. Viable colonies were stained with 0.1% crystal violet and photographed (upper panel). Relative cell density (the lower panel) was obtained from three independent experiments. **C.** Representative colonies of each experimental group are shown. MCF-7 cells transfected with GFP-C1, GFP-PES1 or GFP-PES1 K517R were selected by growth in the presence of G418 (1 mg/ml) for 2 weeks. The cells were then used to prepare the soft agar colony culture. Photographs of the colonies were taken 10 days after seeding. All experiments were repeated at least three times. **D.** MCF-7 cells were transfected with GFP-C1, GFP tagged wild-type or K517R. Flow cytometry analysis was performed after 36 h of growth in the presence of E2 (10 nM). **E.** MCF-7 or T47D cells were stably transfected with GFP-tagged PES1 or K517R together with or without UBC9. The cells were grown in the presence of E2 and then subjected to MTT assay performed according to the manufacturer's instructions. **F.** Comparison of the sizes of tumors formed 30 days after the mice were injected with the tumor cells overexpressing GFP-C1, GFP-tagged PES1 or K517R. (G and H) Tumor images **G.** and tumor weight **H.** were shown. 44 days after the mice were injected subcutaneously with MCF-7 cells overexpressing GFP-C1, GFP-tagged PES1 or K517R. n=5 mice per group in (F, G and H). **I.** The expression of GFP-tagged PES1 or K517R in the tumors extracted from the three groups of mice. ‘*’, *p*<0.05; ‘**’,*p*<0.01.

The role of PES1 SUMOylation in cell proliferation was investigated by determining the effect of PES1 SUMOylation on the proliferation of two breast cancer cell lines. MCF-7 cells overexpressing wild-type PES1 showed more proliferation than those overexpressing K517R, and similar result was observed for T47D cells (Figure 7A). Furthermore, as demonstrated with MCF-7 cells, a greater number of cells appeared when the cells overexpressed wild-type PES1 compared to those that overexpressed K517R (Figure 7B). We next examined the effect of wild-type PES1 or K517R on the colony formation of MCF-7 cells. MCF-7 cells overexpressed GFP-tagged wild-type PES1 showed more proliferation than those that either did not express PES1 or overexpressed K517R (Figure 7C). The effect of PES1 SUMOylation on the process of cell-cycle was also studied. MCF-7 cells that overexpressed wild-type PES1showedan overall increase in the proportion of S-phase cells, with a corresponding decrease in the proportion of cells at the G0/G1 phase compared to the control cells (no overexpression of PES1) (Figure 7D). The proportion of cells in the S-phase also increased when the cells overexpressed PES1 K517R, compared to control cells, but to a lesser extent than those overexpressed wild-type PES1 (Figure 7D). Thus K517R appeared to have a lesser effect on the cell cycle. These data demonstrated that SUMOylation of PES1 could promote the proliferation of breast cancer cells. Overexpression of UBC9 together with wild-type PES1 further promoted the growth of both breast cancer cell lines (Figure 7E). To further identify the role of PES1 SUMOylation in tumorigenesis *in vivo*, nude mice were subcutaneously implanted with MCF-7 cells that stably overexpressed GFP-tagged PES1, PES1 K517R or GFP-C1. Mice implanted with MCF-7 cells that overexpressed GFP-tagged PES1 showed a much larger tumor than mice implanted with MCF-7 cells that overexpressed PES1 mutant or just harbored the control vector during the experimental period (Figure 7F and 7G). Forty four days after the implantation of tumor cells, a 3-fold increase in the weight of the tumor was observed for MCF-7 cells that overexpressed wild-type PES1 compared to tumors derived from cells carried the control vector (Figure 7H). Western blot analysis of the tumors showed that PES1 was successfully expressed (Figure 7I). Overall, these data indicated that wild-type PES1 could promote the growth of cancer cells *in vitro* and *in vivo*.

## DISCUSSION

Posttranslational modification of proteins endows proteins with multiple functions. SUMOylation, an important post-translation modification, manifests its effect through regulating protein-protein interaction, protein stability and protein localization. Although PES1 could be modified by SUMO-1, −2 and −3, the modification was mainly carried out by SUMO-1 (Figure 1), and the modification resulted in improved stability for PES1 by inhibiting its ubiquitination (Figure 3). PES1 is a nuclear protein that is involved in the synthesis and maturation of ribosome. It also has an effect on chromatin stretch. Recent studies have demonstrated that PES1 can promote the development of different tumors [7, 9, 15]. We observed enhancement of ERα stability induced by PES1 SUMOylation, whether with or without E2 treatment, which led to the transactivation of ERα along with the growth of breast cancer cells *in vivo* and *in vitro* (Figure 5–7).

SUMOylation also regulates the stability of proteins. Consistent with this feature was the longer half-life of wild type PES1 compared to PES1 K517R (Figure 3B). Ubiquitin-mediated proteolysis pathway exerts a crucial effect on many cellular physiological functions by regulating different proteins, such as cell surface receptors, transcriptional factors and cofactors. In this study, we showed that PES1 was targeted by ubiquitin and the ubiquitinated level of wild-type PES1 was lower than that of the mutant (Figure 3C). One possible reason could be that PES1 SUMOylation inhibited the interaction between PES1 and the ubiquitin E3 ligase Trim 23, leading to repression of PES1 ubiquitination (Figure 4).

The conjugation of SUMO with a target protein often leads to a change in the conformation of the protein, which then influences its interaction with other proteins. Wild-type PES1 could interfere with the association of ERα with CHIP, while the mutant PES1 K517R almost had no effect. (Figure 5B and 5C). In the absence of E2, CHIP (ubiquitin E3 ligase) constantly targets ERα for its degradation, and PES1 protects ERα from ubiquitination-mediated degradation via inhibiting its interaction with CHIP [9]. However, in the presence of E2, the level of ERα wouldbe reduced through an enhancement of its ubiquitination independent of ChIP [14]. Wild-type PES1 could enhance the stability of ERα with or without E2 treatment (Figure 5A, 5E and 5F), while K517R lost this ability to protect ERα from ubiquitination (Figure 5C). This showed that enhancement of ERα stability by PES1 was dependent on the SUMOylation status of PES1. In the E2-ERα signaling pathway, E2 plays important roles in maintaining the normal function of the mammary gland and it also functions as a potent mammary mitogen [16, 17]. E2 regulates gene transcription mainly through binding with ERα to activate the transcription of ERα-targeted genes, which are involved in cell growth. Enhancement in ERα stability is important to its function. However, E2 can also lead to the loss of ERα via degradation, but the lack of decrease in ERα level in the presence of E2 was a result of E2-stimulated PES1 SUMOylation that effectively maintained the stability of ERα, thereby counteracting the loss of ERα by the direct action of E2. Many studies have tried to understand the effect of ERα on the generation and development of breast cancer, and aberrant ERα signaling is thought to be closely associated with the occurrence of ERα positive breast cancer [18, 19]. Our findings may therefore provide yet a different view on the effect of E2 on ERα-positive breast tumorgenesis. The activation of ERα by E2 is a cycling process, in which E2 promotes the activity of ERα, but at the same time, also promotes the degradation of ERα via increasing its ubiquitination independent of CHIP [14]. The end of the activation cycle is accompanied by the degradation of ERα. In PES1-overexpressed cells, PES1 could rescue ERα from degradation mediated by E2 (Figure 5E, 5F) and this may presumably prolong the transactivation activity of ERα in breast cancer cells. Previous research has shown that PES1 displays high expression in different tumors, including breast cancer [15]. So we proposed that in ERα-positive breast cancer cell, SUMOylated PES1 may protect ERα from ubiquitin degradation whether in the presence or absence of E2, and this protection would promote the growth of ERα-positive breast cancer cells.

The development of cancer is usually caused by aberrant cell cycle. Cell cycle proteins Cyclin D1 controls the transition from the G1 phase to S phase, and it is commonly upregulated in breast cancer cells, leading to defects in mammary development and contributing to the generation of breast cancer, probably by promoting cell growth [20]. Wild-type PES1 promoted the expression of *cyclin D1* in breast cancer cells, and the activation of *cyclin D1* by PES1 was subject to regulation by SUMOylation, since the mutant PES1 that could not be SUMOylated had a weaker effect on the regulation of *cyclin D1* expression (Figure 6). Previous studies have shown that overexpression of PES1 can lead to an increase in the percentage of 32D IRS-1 cell in the G2/M phase [21], whereas knockdown of PES1 can shorten the G2/M phase for colon cancer cells as well as repressing the growth of xenografts [7]. This is consistent with our data, which showed that wild-type PES1 upregulated the expression of *cyclin D1* through maintaining the stability of ERα, and consequently, PES1 positively affected the cell cycle progress and the growth of breast cancer cells.

In summary, we demonstrated in this study that SUMOylation promoted the function and stability of PES1 and identified K517 as the key SUMOylation site. SUMOylation of PES1 also increased the stability of ERα by inhibiting the interaction between ERα and its ubiquitin E3 ligase CHIP, resulting in the inhibition of ERα ubiquitination. The ultimate effect of PES1 SUMOylation at the cell level was the acceleration of cell proliferation and cell cycle progress, as demonstrated for two different breast cancer cell lines. This work has extended our understanding of the mechanism behind PES1-mediated regulation of transcription factors and the consequent promotion of breast cancer cell proliferation. However, much still needs to be explained, such as how PES1 SUMOylation can regulate the stability of ERα in the presence of E2, and whether the promotion of breast cancer cell growth by PES1 SUMOylation is a general phenomenon that also happens in other cancer cells.

## MATERIALS AND METHODS

### Cell culture

COS-7, MCF-7 and T47D cells have been used in our previous study [22, 23]. For the cell starvation assay, MCF-7 and T47D cells were cultured in medium containing 2% charcoal-stripped fetal bovine serum (Gibico) and without phenol red for several days, and then treated with or without E2 (10 nM) for several hours.

### Plasmids, antibodies, si RNA and reagents

Rabbit anti-Myc, anti-Flag, mouse anti-Flag (M2) and cycloheximide (CHX) were obtained from Sigma. Rabbit GFP was purchased from GeneTex. Rabbit anti-PES1and 17β-estrogen (E2) were obtained from Abcam. Rabbit anti-ERα was obtained from Millipore. MG132 was purchased from Merk. The siRNA sequences, siTrim23 and si control, were obtained from GenePharma RNAi Company and their sequences are shown in Supplementary Table S1.

HA-PES1 was obtained from Dr. Dirk Eick (Institute of Clinical Molecular Biology and Tumor Genetics, GSF Research Center, Germany). GFP-PES1 was constructed in our laboratory. The PES1 mutants GFP-K249R, K517R, and 2KR were prepared using the QuikChange Site-Directed Mutagenesis Kit (Stratagene, La Jolla, CA) using HA-PES1 as the template. Myc-tagged SUMO-1, SUMO-2, SUMO-3 along with GFP-SUMO1 and its mutant GFP-SUMO1/GA have been used in our previous studies [11, 12]. Flag-UBC9 was amplified by PCR and subcloned into Flag-pCDNA3.1 vector at *Xho*1 and *Eco*R1 sites.

### Immunoprecipitation and western blotting

Cells harvested from the appropriate culture were lysed and the soluble fraction of the cell extract was subjected to western blot and immunoprecipitation were conducted as previously described [11, 24]. Clean Blot IP Detection Reagent was purchased from Thermo Scientific.

### Immunofluorescence staining

MCF-7 cells were transfected with GFP-tagged wild-type or mutant PES1 K517R for 36 h, and the cells were then subjected to immunofluorescence staining assay as previously described [11].

### In vitro SUMOylation assay

The vitro SUMOylation assay was performed using a SUMOylation Kit purchased from Enzo Life Sciences (NY, USA). In brief, purified GST-PES1 was incubated in a mixture containing recombinant E1, Ubc9 (E2), and Mg-ATP with or without SUMO-1 for 1 h at 30°C according to the manufacturer's instructions. The reaction was terminated with SDS-PAGE loading buffer.

### Luciferase reporter assay

MCF-7 and T47D cells transfected with the appropriate plasmids for 24 h were starved for 2 days followed by stimulation with or without E2 (10 nM) for another 16 h. The cells were then subjected to luciferase assay using a dual luciferase Kit (Promega, USA).

### RNA extract and reverse transcription-PCR

MCF-7 cells were stimulated with or without E2 (10 nM) for 16 h after they had been starved for 2 days. Total RNA was extracted as previously described [22]. The primers used for the RT-PCR analysis of *cyclin D1* and *GAPDH* were as previously described [22]. C*yclin D1* mRNA level was normalized with the *GAPDH* mRNA level.

### Cell proliferation assays

MCF-7 and T47D cells were stably transfected with GFP-C1, GPF-PES1 or GFP-K517R. The cells were starved for 1 day, and then stimulated with E2 (10 nM) for several days. MTT assay was performed using an MTT assay kit obtained from KeyGEN BioTECH (Nanjing, China). For cell cycle analysis, MCF-7 cells transfected with GFP-C1, GPF-PES1 or GFP- K517R were cultured in the presence of E2 (10nM) for 36 h, and then subjecting to flow cytometry analysis according to the protocol as previously described [22]. For crystal violet staining assay, MCF-7 cells transfected with GFP-C1, GPF-PES1 or GFP-PES1 K517R were maintained in DMEM (containing 10% fetal bovine serum) medium containing G418 (1 mg/ml) for 2 weeks. About 5 × 10^3^ cells/well were then plated in 6-well plate and grown in the presence of E2 (10 nM) for 7 days. After that the cells were subjected to crystal violet staining according to **a** previous report [23].

### Soft-agar colony culture

MCF-7 cells were transfected with wild-type PES1, K517R or GFP-C1 and grown in DMEM (containing 10% fetal bovine serum) medium containing G418 (1 mg/ml) for 2 weeks. The cell suspension containing 2000 cells/group were used for soft-agar colony culture assay as previously described [25, 26].

### Human breast cancer xenograft model

About 1 × 10^7^ MCF-7 cells stably transfected with GFP-PES1, GFP-PES1 K517R or GFP-C1 were resuspended in 50% Corning® Matrigel® Basement Membrane Matrix High Concentration (Corning) in a final volume of 100 μl, and then injected into the left flank of five to six week-old female athymic nude mice (BALB/c mice, from Animal Experiment Center of Dalian Medical University). All mice were maintained under specific pathogen-free (SPF) conditions. All experiments involving animals were performed under the regulations set by the Ethics Committee for Dalian University of Technology. Tumor growth was facilitated by feeding the animals with water containing E2 at a concentration of 1 mg/L [27]. After 44 days, the mice were killed in a humane manner as previously described [28].

### Statistical analysis

All statistical analyses of data were performed with ANOVA. Data were given as means ± SDs, and statistical significance was considered at either *P* value <0.05 or 0.01 level.

## SUPPLEMENTARY MATERIALS TABLE


