# Supplementary material for: SUMOylation of PES1 upregulates its stability and function via inhibiting its ubiquitination
Source: Oncotarget. 2016 Jul 8;7(31):50522–34. doi: 10.18632/oncotarget.10494 (PMC5226600; doi:10.18632/oncotarget.10494)
Supplement: Supplementary file 1 [file oncotarget-07-50522-s001.pdf]

SUMOylation of PES1 upregulates its stability and function via inhibiting its ubiquitination

SUPPLEMENTARY TABLE

Supplementary Table S1: Sequences for Si Trim23 and Si control

Primer name	Sequence
Si Trim23-168 sense	GCUAGAGUGUGGAGUUUGUTT
Si Trim23-168 antisense	ACAAACUCCACACUCUAGCTT
Si Trim23-299 sense	GCCCAUUUGAUCGACAAGUTT
Si Trim23-299 antisense	ACUUGUCGAUCAAAUGGGCTT
Si Trim23-1377 sense	GCCCAUUCCAACAAUUGGUTT
Si Trim23-1377 antisense	ACCAAUUGUUGGAAUGGGCTT
Si control sense	UUCUCCGAACGUGUCACGUTT
Si control antisense	ACGUGACACGUUCGGAGAATT
